# Immunization against Typhoid by Gastro-Intestinal Tract

**Published:** 1915-05

**Authors:** 


**Immunization Against Typhoid by Gastro-Intestinal Tract.** Paris and Desmouliere, Bull. et Mem. de la Soc. de Med. de Paris, Feb. 12, 1915. M. H. Vincent, Presse Med., Nov. 19, 1914, in a personal study with review of the work of the English Commission of 1913, concludes that this method is inefficient. The present authors, from personal tests, agree. Both experienced a slight febrile reaction and one was considerably jaundiced.

